# Hypoxia Response Element-Directed Expression of aFGF in Neural Stem Cells Promotes the Recovery of Spinal Cord Injury and Attenuates SCI-Induced Apoptosis

**DOI:** 10.3389/fcell.2021.693694

**Published:** 2021-06-14

**Authors:** Yibo Ying, Yifan Zhang, Yurong Tu, Min Chen, Zhiyang Huang, Weiyang Ying, Qiuji Wu, Jiahui Ye, Ziyue Xiang, Xiangyang Wang, Zhouguang Wang, Sipin Zhu

**Affiliations:** ^1^Department of Orthopaedics, The Second Affiliated Hospital and Yuying Children’s Hospital of Wenzhou Medical University, Wenzhou, China; ^2^Molecular Pharmacology Research Center, School of Pharmaceutical Science, Wenzhou Medical University, Wenzhou, China; ^3^Department of Pain Medicine, The Second Affiliated Hospital and Yuying Children’s Hospital of Wenzhou Medical University, Wenzhou, China

**Keywords:** spinal cord injury, acidic fibroblast growth factor, adeno-associated virus, neural stem cell, endoplasmic reticulum stress, apoptosis

## Abstract

Reducing neuronal death after spinal cord injury (SCI) is considered to be an important strategy for the renovation of SCI. Studies have shown that, as an important regulator of the development and maintenance of neural structure, acidic fibroblast growth factor (aFGF) has the role of tissue protection and is considered to be an effective drug for the treatment of SCI. Neural stem cells (NSCs) are rendered with the remarkable characteristics to self-replace and differentiate into a variety of cells, so it is promising to be used in cell transplantation therapy. Based on the facts above, our main aim of this research is to explore the role of NSCs expressing aFGF meditated by five hypoxia-responsive elements (5HRE) in the treatment of SCI by constructing AAV–5HRE–aFGF–NSCs and transplanting it into the area of SCI. Our research results showed that AAV–5HRE–aFGF–NSCs can effectively restore the motor function of rats with SCI. This was accomplished by inhibiting the expression of caspase 12/caspase 3 pathway, EIF2α–CHOP pathway, and GRP78 protein to inhibit apoptosis.

## Introduction

Spinal cord injury (SCI) is a catastrophic traumatic illness in the central nervous system (CNS), which can lead to impaired movement, sensation, and other functions (Ahuja et al., 2017; Chhabra and Sarda, 2017; Bradbury and Burnside, 2019; Song et al., 2019). It is estimated that 2.5 million people worldwide suffer from SCI, and over 130,000 new cases are reported each year (Thuret et al., 2006). Due to the non-regeneration of the CNS, SCI may lead to permanent functional damage (Sofroniew, 2018). Apoptosis can result in the loss of numerous neurons, which contributes to the sensory and motor function loss (Crowe et al., 1997; Beattie, 2004; Abbaszadeh et al., 2020; Shi et al., 2021). Therefore, it is of great significance to take apoptosis as the target for the renovation of SCI.

At present, the treatment of acute SCI is limited to surgery and hormone therapy, and its clinical effect is not ideal (Ramer et al., 2014). Studies have shown that the CNS is very difficult to repair, and scientists hope that advances in stem cell research may eventually restore neurocirculation in people with SCI, putting the hope of future treatment on stem cells (Bourzac, 2016). NSCs isolated from the developing CNS and peripheral nervous system have aroused increasing interest of researchers (Peruzzotti-Jametti et al., 2018; Deng et al., 2019). NSCs have the ability to self-replace and differentiate into a variety of cells (Navarro Negredo et al., 2020). Some experiments have shown that after transplantation, most of the NSCs differentiate into corresponding cells and migrate and integrate into the host tissue due to the regulation of signal factors (Denoth-Lippuner and Jessberger, 2021). Thus, the neural loop is reconstructed, and the relay station between the regenerated axons of the host and the denervated neurons is provided (Xu et al., 2011; Li et al., 2020; Zheng et al., 2020). As an immune exemption organ, the CNS has almost no rejection in neural stem cell transplantation between different individuals and even between different species (Xu et al., 2011). Therefore, NSCs are a good choice for stem cell transplantation in the treatment of SCI.

AFGF has been proved to be an important molecule in the growth and development of spinal cord (Tsai et al., 2006; Kuo et al., 2011; Wang et al., 2018). AFGF plays a significant role in expressing mitosis and pluripotent activity, which is closely related to the development and maintenance of neural structure (Nurcombe et al., 1993). Some studies have shown that aFGF has a tissue-protective effect and can promote the repair of SCI (Eckenstein et al., 1991; Maquet et al., 2001; Wang et al., 2017). However, previous studies have shown that the effect of using conventional methods to apply aFGF to the site of SCI is not ideal. The possible reason is that direct injection has the problems of short half-life and easy degradation. Therefore, the main purpose of this study is to improve the concentration of aFGF in the injured area by combining regulated aFGF with transplanted NSCs, so as to achieve the purpose of repairing SCI.

Adeno-associated virus (AAV) vectors are the main platform for *in vivo* gene therapy delivery. AAV is safe and can deliver its single-stranded DNA (ssDNA) vector genome to various tissues and cell types, and can be expressed for a long time (Wang et al., 2019, 2020). Hypoxia-response element (HRE) is the main regulator of cell response to hypoxia, which can regulate the transcription of specific genes under hypoxia, so it is widely used in the construction of hypoxia-regulated expression genes (Wu et al., 2015; Zhu et al., 2021). In this study, we used AAV with HRE, to induce HRE-mediated expression of aFGF of NSCs in hypoxic environment, which effectively reduced the tumorigenicity and short half-life emulated with direct injection of aFGF. Thus, AAV–5HRE–aFGF–NSCs is brought about and transplanted to the original injured site of SCI. We used video camera recording (VCR), inclined plate test, footprint analysis, and Basso–Beattie–Bresnahan (BBB) score scale to study the role of AAV–5HRE–aFGF–NSCs in the restoration of locomotion in SCI rats. In order to study the regulation of endoplasmic reticulum (ER) stress-induced apoptosis, we detected the expression of caspase 12–caspase 3, EIF2α–CHOP pathway, and GRP78. Our research shows that AAV–5HRE–aFGF–NSCs can promote the repair of SCI by inhibiting ER stress.

## Materials and Methods

### Reagents and Antibodies

The primary antibodies, including aFGF (ab169748), NeuN (ab177487), caspase 12 (ab62484), caspase3 (EPR18297), EIF–2α (ab169528), CHOP (ab11419), and GRP78 [EPR4041 (2)] and the secondary antibodies: goat anti-rabbit 488 (ab150077), goat anti-mouse 488 (ab150113), goat anti-rabbit (HRP) (ab6721), and rabbit anti-mouse (HRP) (ab6728) were all bought from Abcam. The DAPI is also provided by Abcam (MC, United Kingdom).

### Isolation and Culture of Neural Stem Cells

The cerebral cortex of SD rat embryos of 14–16 days were separated and immersed in D-Hanks solution. We carefully removed the cerebrovascular and meninges under the microscope. After trimming, the tissue was digested in trypsin (0.125%) and ethylenediaminetetraacetic acid (0.102%). After terminating the digestion with complete medium, we centrifuged the liquid at 1,000 rpm for 3 min and collected the cell suspension.

The components of neural stem cell culture medium (100 mL) were as follows: DMEM 96 mL, B27 2 mL, glutamine 1 mL, horse serum 1 mL, glutamine 2 mmol/L, penicillin, heparin 100 μL, basic fibroblast growth factor (bFGF) 20 μL, and epidermal growth factor (EGF) 10 μL cultured in 5% CO_2_ and 37°C humidified incubator.

### Polylysine Coating of Neural Stem Cells

The polylysine solution was diluted to 0.1 mg/mL with sterile water. Before use, the diluted polylysine solution was placed in a room, and the temperature was set to room temperature. Then, the slide was immersed in the diluted polylysine solution for 5 min. After completion, it was dried overnight at room temperature. Before immunofluorescence staining, the cell suspension was dropped on the glass slide and cultured in a 37°C incubator.

### *In vitro* Scratch Motility Assay

All the cells were randomly divided into four groups, including Sham, thapsigargin (TG), 4-phenylbutyric acid (4–PBA), AAV–5HRE–NSCs, and AAV–5HRE–aFGF–NSCs group. All cells are fixed to the well plate with polylysine. The doses of Tg and 4–PBA were 100 and 500 nM, respectively.

A 200-μL tip was utilized to draw a linear scratch on the cell monolayer to generate a cell-free area. After that, the cells were rinsed twice in PBS. At 0, 12, and 24 h, the wound was analyzed and photographed by an inverted microscope. After exporting the data, the injured area was analyzed by Image J.

### Virus Construction

We used the H4409 AAV–5HRE–CMVmp vector (Obio, Shanghai, China) and then introduced aFGF into the vector through the seamless connection kit (Obio, Shanghai, China) to construct AAV–5HRE–aFGF. It was tested whether stocks of AAV–5HRE–aFGF and AAV–5HRE contain virus particles with a titer of 2.56 × 10^12^ and 2.32 × 10^12^/mL, respectively.

### Cell Transformation

After that, NSC was transduced with the multiplicity of infection of 1 × 10^5^ AAV–5HRE–aFGF and AAV–5HRE virus particles, reaching a transduction rate of 90%. The transduced cells were cultured under hypoxia (<1% O_2_) conditions for at least 6 h.

### Spinal Cord Injury Model

Adult female SD rats (48) were randomly separated into four groups: Sham, SCI, AAV–5HRE–NSCs, and AAV–5HRE–aFGF–NSCs groups. The rats were placed in 5% isoflurane (220–250 g) till they lost consciousness, and then they were continuously anesthetized with 3% isoflurane. The skin of SD rats was cut along the middle of the back, and after the spinous process and lamina process of T9–T10 were cut, the spinal cords were exposed. After the midline as the center, a 10-g hammer was utilized to hit T9 segment from 25-mm high to produce acute SCI injury. The sham group went through the operation similarly without SCI by collision. The rats were given bladder massage every morning and evening to help them urinate.

### Cell Transplantation

The extracted NSCs were transduced in advance with 1 × 10^5^ multiplicity of infection of AAV–5HRE–aFGF or AAV–5HRE virus particles for over 48 h. Seven days past SCI, another operation was performed to expose the injured spinal cord. NSCs, 5 × 10^5^ were resuspended in 10 μL at 0–4°C PBS were syringed into the SCI center (depth: 1 mm) through a stereotaxic device and a micro syringe pump. After transplantation, the mice were injected intraperitoneally (i.p.) with cyclosporine (10 mg/kg dose) for immunosuppression, which was continued every day after transplantation (Yoo et al., 2014).

### Western Blot Analysis

For protein analysis, tissues were taken from the upper and lower 0.5 cm of the SCI area and quickly stored in −80°C for Western blotting analysis. The tissue was homogenized in a modified radioimmunoprecipitation assay (RIPA) buffer (25 mM Tris-HCl, 150 mM NaCl, 1% Nonidet Pmur40, 1% sodium deoxycholate, and 0.1% SDS). The extract was quantitatively analyzed by dicarboxylic acid (BCA) reagent (Thermo, Rockford, IL, United States). Gel, 11.5%, was configured, and 50 μg of protein was placed in it. Then, it was transferred to a PVDF membrane (Bio-Rad, Hercules, CA, United States). The membrane was blocked with 5% milk (Bio-Rad) in TBS containing 0.05% Tween 20 for 1 h. The membrane was incubated in anti-aFGF antibody and placed in a refrigerator at 4°C overnight. The membrane was washed in TBS three times and incubated with horseradish peroxidase-coupled secondary antibody at room temperature for 1 h. The experimental results were visualized through the ChemiDocXRS^TM^ imaging system (Bio-Rad), and the strip density was quantitatively measured using the Multigauge software of 2006 Science Lab (FUJIFILM Corporation, Tokyo, Japan). We used the analyzing Software, Quantity One (Bio-Rad) to analyze the relative density of stripes and use GAPDH to normalize them.

### Locomotion Assessment

In order to evaluate the motor function after SCI, two trained researchers used the Basso–Beattie–Bresnahan (BBB) scale for behavior analysis. They knew the scoring criteria well, but did not understand the experimental conditions. Scores range from 0 (lower limb paralysis) to 21 (responding to normal locomotion). The inclined board test is the maximum angle at which rats cannot fall for 5 s through equipment, testing, and recording. Footprint analysis was carried out by soaking the hind feet of rats with red dye and making them climb over a frame of appropriate size (1 m × 7 cm). Then results were scanned and processed.

### Video Imaging of Locomotion

Six rats were randomly selected from the Sham group, SCI group, AAV–5HRE–NSCs group, and AAV–5HRE–aFGF–NSCs group. Using the camera (Leica), each group of rats walked on a 1-m-long glass track with markings on their hind legs to take pictures of the hips, knees, ankles, and feet and estimate their positions. The exercise of rats was evaluated by the following parameters: (1) weight support (height; the height of the torso from the ground), (2) leg extensor spasm (quantified by the time the foot was overstretched and dragged and the leg staying on the back), (3) the number of footprints (the number of previous steps as a reference), and (4) the posture of the foot (the distance between the beginning of the ankle and the rear of the hip). Pace was determined through the front legs (steps in the front legs per second) (Li et al., 2017).

### Hematoxylin and Eosin Staining and Nissl Staining

In order to obtain the samples, rats with SCI for 60 days were anesthetized with the 1% pentobarbital (40–50 mg/kg, intraperitoneal injection). After thoracotomy, the whole body was perfused with 500 mL of 0.9% NaCl and then 100 mL of paraformaldehyde phosphate buffer solution. The spinal cord at the level of T8–10 was removed and spent the night in 4% paraformaldehyde. After paraffin embedding, the paraffin sections (5 μm) were stained in hematoxylin and eosin (H&E) and Nissl for further evaluation.

### Immunohistochemistry

Spinal cord slices were incubated in 80% methanol and 3% H_2_O_2_ for 30 min, and then placed in a closed solution at 25°C for 1 h. The sample and the first antibody were then incubated overnight in a refrigerator at 4°C: caspase 12 (1:4,000), caspase 3 (1:500), Eif–2 α (1:100), CHOP (1:200), and GRP78 (1:200). After washing with PBS three times, the second antibody bound to horseradish peroxidase was incubated at 37°C for 2 h. We terminated the reaction by 3pyr3-diaminobenzidine (DAB). Finally, the nucleus is stained with hematoxylin. Six regions were randomly selected in each sample, and the densities of caspase 12, caspase 3, Eif–2 α, CHOP, and GRP78-positive neurons were counted. We display the results through Nikon ECLPSE 80i (Nikon, Tokyo, Japan).

### Immunofluorescence Staining

When finished with dewaxing, rehydration, and antigen retrieval, all the slices were incubated at 37°C in PBS containing 5% bovine serum albumin (BSA) and 0.1% Triton X–100 for 1 h. The slices were incubated with suitable primary antibody at 4°C overnight and stained with DAPI (0.25 μg/mL) dye to observe the nucleus. In order to evaluate neurons and aFGF, we used anti-NeuN (1:200) and anti-aFGF (10 μg/mL) antibodies, respectively. After incubation, the slices were then washed with PBS three times at room temperature, and the samples were incubated with the secondary antibody (1:500) at 37°C for 1 h. When finished, the slices were washed with PBS three times for 5 min each time. All pictures were shot with Nikon ECLIPSE Ti microscope (Nikon, Tokyo, Japan) and counted with ImageJ software, and then analyzed with Prism 7 (GraphPad, San Diego, CA, United States) to generate statistical charts.

### Statistical Analysis

Statistically, the data are expressed as the average ± SD. When comparing the two groups of data, the double-sided Student *t*-test was carried out. When there are more than two sets of data, Dunnett and the ex-post test of one-way analysis of variance (ANOVA) were utilized to assess the data. *P* < 0.05 indicates significant difference.

## Results

### Expression of Adeno-Associated Virus–Five Hypoxia-Responsive Elements–Acidic Fibroblast Growth Factor in Neural Stem Cells

**FIGURE 1:**
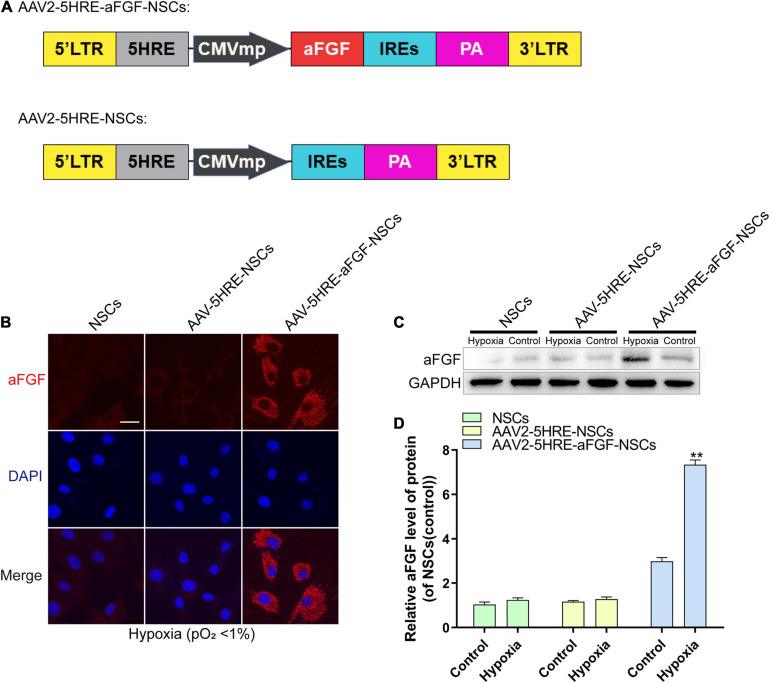
The preparation and identification of AAV–5HRE–aFGF–NSCs. **(A)** The schematic diagrams of the vector construction: AAV–5HRE–aFGF and AAV–5HRE. **(B)** The immunofluorescence shows the expression of aFGF (red) and DAPI (blue) in different groups. Magnification: 40×; Scale: 50 μm. **(C)** Western blotting showing the expression of aFGF in each group. **(D)** The quantitative analysis of aFGF protein expression. **P < 0.01. Data are represented as mean ± SD (*n* = 6). AAV, adeno-associated virus; 5HRE, five hypoxia-responsive elements; aFGF, acidic fibroblast growth factor; NSCs, neural stem cells.

In order to detect whether AAV-mediated aFGF was directly regulated by HRE under hypoxia, AAV–5HRE–aFGF was structured and transduced into NSCs (Figure 1A). From the immunofluorescence staining, it could be seen that AAV–5HRE–aFGF in nerve cells successfully expressed aFGF under hypoxia (Figure 1B). In addition, the level of aFGF protein was evaluated by Western blotting. We found that under normoxia, the expression of aFGF was weak, while the expression of aFGF of AAV–5HRE–aFGF–NSCs under hypoxia was significantly higher than that of other groups (Figures 1C,D). All our findings showed that AAV–5HRE–aFGF was successfully transfected into NSCs, while aFGF was expressed only under hypoxia.

### Expression of Acidic Fibroblast Growth Factor in Adeno-Associated Virus–Five Hypoxia-Responsive Elements–Acidic Fibroblast Growth Factor–Neural Stem Cells Group *in vivo*

**FIGURE 2:**
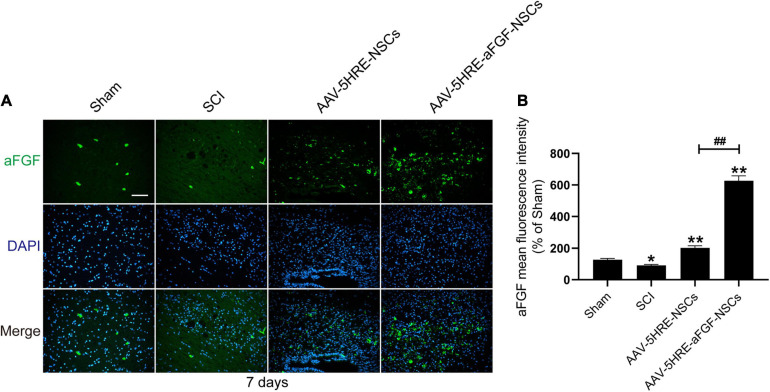
Detection of aFGF production *in vivo.* **(A)** The immunofluorescence shows the expression of aFGF (green) and DAPI (blue) in different groups. Magnification: 20×; Scale: 100 μm. **(B)** Mean fluorescence intensity of aFGF. ##,*P < 0.05. **P < 0.01. Data are represented as mean ± SD (*n* = 6).

To study the expression of aFGF after transplantation of AAV–5HRE–aFGF–NSCs, we performed immunofluorescence staining of aFGF protein on tissue samples for 14 days. The results showed that the positive rate of aFGF in the AAV–5HRE–aFGF–NSCs group was significantly higher than the positive rate in the other groups, and the positive rate in the injured area was much higher than the positive rate in the non-injured area (Figure 2). In summary, AAV–5HRE–aFGF–NSCs expressed high levels of aFGF *in vivo* and were regulated by hypoxia.

### Adeno-Associated Virus–Five Hypoxia-Responsive Elements–Acidic Fibroblast Growth Factor–Neural Stem Cells Promotes the Recovery of Motor Function to a Great Extent

**FIGURE 3:**
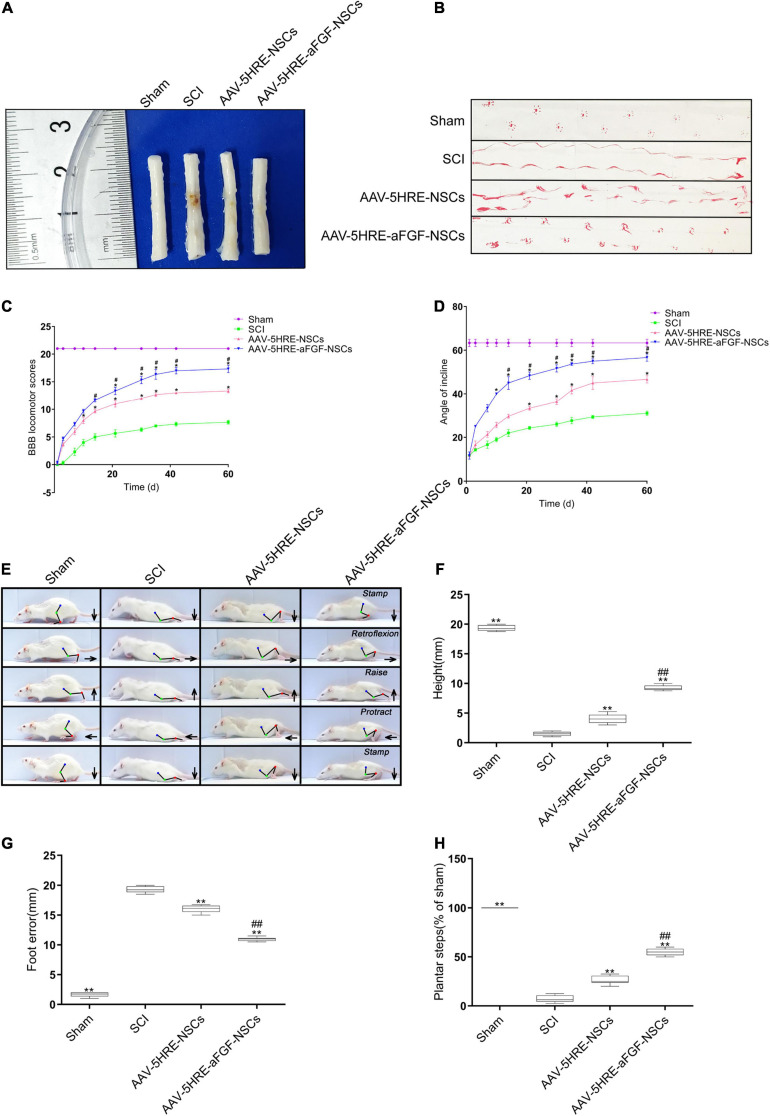
AAV–5HRE–aFGF–NSCs enhance the recovery of motor function in SCI rats. **(A)** Image showing varying degrees of damage of spinal cords in different groups. **(B)** Footprint analysis of the Sham, SCI group, AAV–5HRE–NSCs group, and AAV–5HRE–aFGF–NSCs group in 30 d.a.t. **(C)** The BBB scores in the Sham, SCI, AAV–5HRE–NSCs, and AAV–5HRE–aFGF–NSCs rats. **(D)** The inclined plane test scores of rats in the Sham, SCI group, AAV–5HRE–NSCs group, and AAV–5HRE–aFGF–NSCs group. **(E)** Images from videos showing rats’ walk in 30 d.a.t. The weight support, slow steps, leg extensor spasms, and foot placement were processed. The direction of the foot movement is marked by arrows. The initial position of the move is highlighted by red dots. **(F)** Weight support. **(G)** Foot error. **(H)** The spasm. #,*P < 0.05. ##,**P < 0.01. All the data are represented as mean ± SD (*n* = 6). SCI, spinal cord injury; BBB, Basso–Beattie–Bresnahan.

AAV–5HRE–aFGF–NSCs treatment could reduce nerve death after SCI and enhanced the restoration of motor function in SCI rats. In order to evaluate the effect of the therapy of AAV–5HRE–aFGF–NSCs on SCI rats, we took visual images of spinal cord morphology. Compared with the large area blackening of the spinal cord of SCI group, the blackening degree in the AAV–5HRE–aFGF–NSCs group was particularly lower, showing the appearance of the SCI group closest to the Sham group (Figure 3A). Through footprint analysis, compared with the SCI group, the motor function of the AAV–5HRE–aFGF–NSCs group was improved (Figure 3B). Similarly, through the BBB score and the inclined plate test, the AAV–5HRE–aFGF–NSCs group showed significant recovery (Figures 3C,D). In addition, the video camera recording also showed that the foot error index of the AAV–5HRE–aFGF–NSCs group was lower, while the height and plantar step index were higher, which indicated that the functional recovery was better (Figure 3E). In addition, compared with the SCI group, the foot position error of the AAV–5HRE–aFGF–NSCs group decreased, and the height of the torso on the ground and the number of plantar steps increased (Figures 3F–H).

### Adeno-Associated Virus–Five Hypoxia-Responsive Elements–Acidic Fibroblast Growth Factor–Neural Stem Cells Improves Neuronal Regeneration and Promotes the Repair of Spinal Cord Injury

**FIGURE 4:**
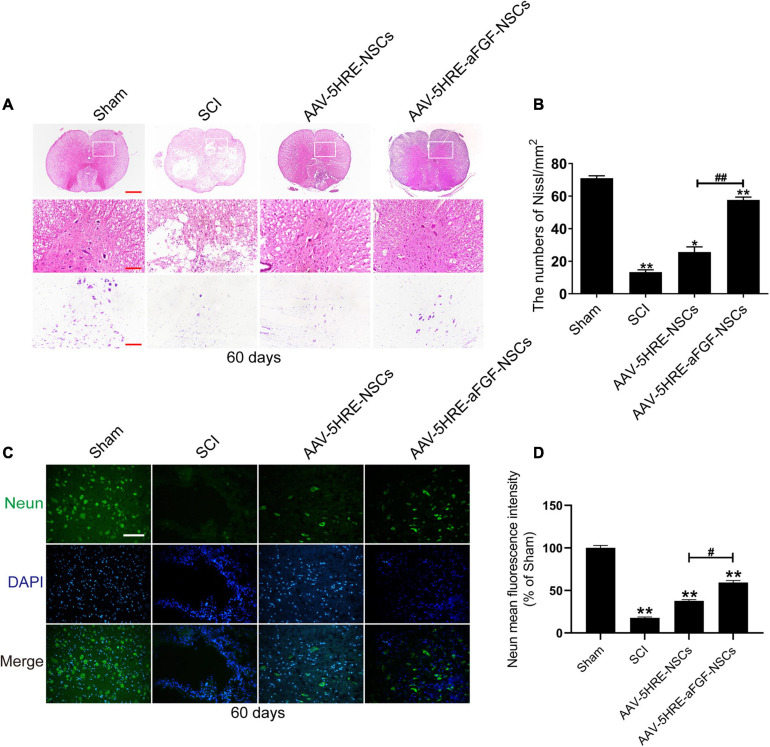
AAV–5HRE–aFGF–NSCs treatment improved the structural integrity of the spinal cord and the restoration of neurons. **(A)** Images of whole cross section after hematoxylin and eosin (H&E) staining and Nissl staining. Magnification: 4×; Scale: 500 μm. The scale bar of Nissl staining images are 100 μm. **(B)** Quantification of Nissl staining in different groups. *P < 0.05. **P < 0.01. Data are represented as mean ± SD (*n* = 6). **(C)** Immunofluorescence to show the expression of NeuN (green) and DAPI (blue) in different groups. Magnification: 40×; Scale: 50 μm. **(D)** Mean intensity of fluorescence of NeuN in different groups. #,*P < 0.05. ##,**P < 0.01. Data are represented as mean ± SD (*n* = 6).

After 60 days of contusion, the spinal cord samples were examined by cross-sectional Nissl staining and H&E staining to evaluate the structure of spinal cord tissue. Emulated with the Sham group, some tissues in the SCI group were harmed, accompanied by a significant decline in the quantity of neurons and the formation of cavities. In the AAV–5HRE–aFGF–NSCs group, there were a few gaps, but no obvious necrosis, showing a more complete tissue structure (Figures 4A,B). From immunofluorescence staining of NeuN, we knew that the fluorescence signal density of the AAV–5HRE–aFGF–NSCs-treated group was even higher than that of the SCI group, which revealed a fine recovery of the nerves (Figures 4C,D). This showed that AAV–5HRE–aFGF–NSCs plays a positive role in axonal survival and regeneration.

### Adeno-Associated Virus–Five Hypoxia-Responsive Elements–Acidic Fibroblast Growth Factor–Neural Stem Cells Inhibits Endoplasmic Reticulum Stress-Induced Apoptosis and Enhances the Restoration of Spinal Cord Injury

**FIGURE 5:**
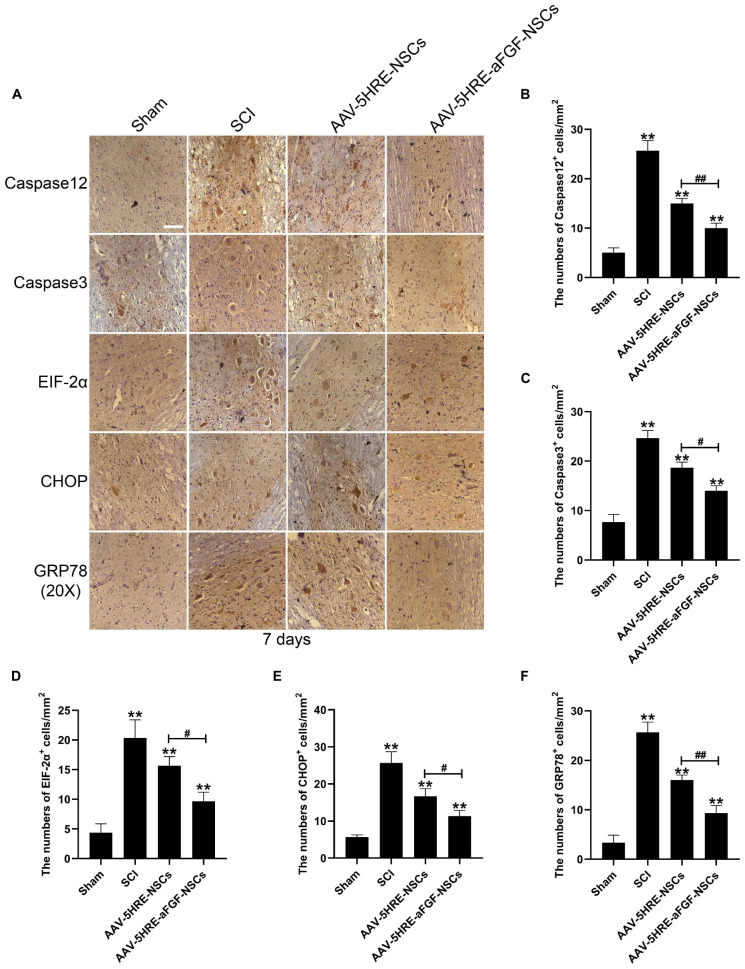
The regulation signals are significant for the neuroprotective effect of AAV–5HRE–aFGF–NSCs. **(A)** Immunohistochemistry for caspase 12, caspase 3, EIF–2α–CHOP, and GRP78 in the Sham, SCI, AAV–5HRE–NSCs, and AAV–5HRE–aFGF–NSCs group. Magnification: 20×; Scale: 100 μm. **(B–F)** Analysis of immunohistochemistry positive cells. **p < 0.01. ^#^*p* < 0.05. ^##^*p* < 0.01. Data are represented as mean ± SD (*n* = 6).

In order to figure out if AAV–5HRE–aFGF–NSCs promotes the recovery of SCI by inhibiting apoptosis that was induced by ER stress, we detected the caspase 12–caspase 3 pathway, EIF2α–CHOP pathway, and GRP78 expression of ER stress (Figure 5) by immunohistochemical staining. As is shown in the figure, emulated with the SCI group, the AAV–5HRE–NSCs group could reduce the expression level of caspase 12. However, the decrease in the AAV–5HRE–aFGF–NSCs group was greater than the decrease in the AAV–5HRE–NSCs group. The caspase 3 expression level in the AAV–5HRE–NSCs group was even lower than the expression level in the SCI group. At the same time, the production of caspase 3 in the AAV–5HRE–aFGF–NSCs group was more significantly inhibited. This suggests that AAV–5HRE–aFGF–NSCs could further inhibit caspase 12–caspase 3–mediated apoptosis in SCI, thus, promoting the recovery of SCI. In the determination of EIF–2α, although there was a significant decrease in the AAV–5HRE–NSCs group compared with the SCI group, the degree of the decrease in the AAV–5HRE–aFGF–NSCs group was even obvious. Similarly, emulated with the SCI group, the AAV–5HRE–NSCs group could reduce the expression of CHOP, but the decrease in the AAV–5HRE–aFGF–NSCs group was more significant. Therefore, in the EIF–2α/CHOP pathway, treatment of NSC alone could also reduce the expression of EIF–2α and CHOP. Compared with the AAV–5HRE–NSCs group, the therapeutic effect of AAV–5HRE–aFGF–NSCs was better. Finally, we also detected the expression of GRP78 in each group. We observed that although the level of GRP78 in the AAV–5HRE–NSCs group was still better than the level in the SCI group, the improvement in the AAV–5HRE–aFGF–NSCs group was more significant. To sum up, through the treatment of AAV–5HRE–aFGF–NSCs, the levels of caspase 12–caspase 3 pathway, EIF2α–CHOP pathway, and GRP78 induced by ER stress decreased, which effectively inhibited the apoptosis and finally enhanced the recovery of SCI.

### Adeno-Associated Virus–Five Hypoxia-Responsive Elements–Acidic Fibroblast Growth Factor Increases the Migration and Restoration Ability of Neural Stem Cells by Inhibiting Endoplasmic Reticulum Stress

**FIGURE 6:**
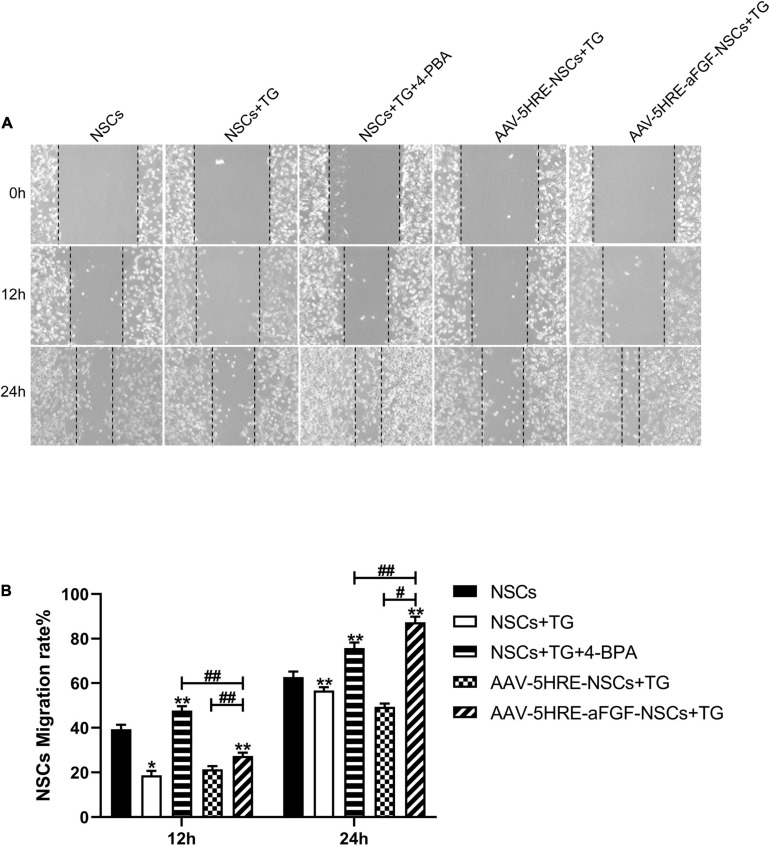
AAV–5HRE–aFGF–NSCs promote NSC migration by inhibiting endoplasmic reticulum (ER) stress. **(A)** Cell migrating images in control, TG, TG^+^ 4–PBA, TG^+^ 4–BPA, AAV–5HRE–NSCs, and AAV–5HRE–aFGF–NSCs group. These images are 0-, 12-, and 24-h inspection images taken by an inverted phase-contrast microscope. **(B)** The NSCs migration rate for 12 and 24 h. ^#^, *P < 0.05. ##, **P < 0.01. The data were processed by ImageJ and presented as the mean values ± SD, *n* = 6. TG, thapsigargin; PBA, phenylbutyric acid.

The results of the cell migration test in different groups are showed at 0, 12, and 24 h after scratch (Figure 6), respectively. With the passage of time, the acellular area of each group declined. Emulated with the control group, the acellular area in the TG group was larger, which indicated that apoptosis had an effect and inhibited cell migration. Compared with the TG group, the wound area of the AAV–5HRE–aFGF–NSCs group was significantly smaller, and the migration speed was faster. This result is the same as the recovery trend of the 4–PBA group, but the migration rate is higher, which indicates that AAV–5HRE–aFGF–NSCs can inhibit cell apoptosis more effectively than 4–PBA.

## Discussion

Due to the non-renewability of the CNS, it is difficult to regenerate neurons through its own repair (McDonald and Sadowsky, 2002; Tran et al., 2018). Therefore, exogenous neurons are needed to supplement the missing parts. Current research is mainly focused on stem cell transplantation (Desgres and Menasché, 2019; Lindsay et al., 2020). NSCs are able to self-replace and differentiate into a variety of cells (Kumamaru et al., 2018). After transplantation, most NSCs can differentiate and migrate into the host tissue, providing an important supplement for the loss of neurons (Assinck et al., 2017). Literature has shown that transplanted NSCs also have the capability to secrete neurotrophic factors, which can protect neurons (Fan et al., 2018). In the research of this article, we found that the effect of neural stem cell therapy alone is not good, and it needs to be combined with other treatments to achieve better results.

AFGF has been proven to play a significant role in neuroprotection and axon regeneration, and its feasibility has been verified in clinical trials (Lee et al., 2011). Experiments have confirmed that aFGF has a good neurotrophic effect. At present, the research on aFGF is mostly focused on promoting the growth and differentiation of neurons and neurotrophic effects, but its role in ER stress-induced apoptosis is still unknown (Garré et al., 2010; Kuo et al., 2011). Moreover, as a macromolecular protein, it is easy to decompose in the body and has a short half-life, so a carrier is needed to solve this problem (Zakrzewska et al., 2008; Haenzi and Moon, 2017).

Although scientists have made considerable progress in the research of SCI, single-factor research has great limitations in the repair of SCI (Fouad et al., 2021). Therefore, the organic combination of aFGF and NSCs to achieve a better repair effect on SCI is an important goal of our current research.

In recent years, AAV has been widely used in neuroscience research (Leborgne et al., 2020; Wang et al., 2020). Emulated with adenovirus and lentivirus, AAV is rendered with more stable expression ability and higher immunocompatibility in nerve cells (Li and Samulski, 2020). The HRE can induce the expression of specific genes under hypoxic conditions (Schörg et al., 2015; Zhang et al., 2016). We used AAV to carry 5HRE–aFGF into NSCs and achieved a high expression of aFGF induced by hypoxia. In this study, we built AAV–5HRE–aFGF–NSCs and transplanted them to the SCI, and verified their repairing effect on the locomotion of the SCI rats.

In order to further explore its role after SCI, we focused our attention on apoptosis. Apoptosis, as one of the four types of programmed death, is activated after cells are damaged by the outside world, and induces the autonomous and orderly death of cells through the activation of specific pathways (Bedoui et al., 2020). After SCI, ischemia and hypoxia can impair the function of the ER, causing the accumulation and aggregation of unfolded proteins (Hetz, 2012; Cybulsky, 2017; Hetz and Saxena, 2017; Ren et al., 2021). This condition is ER stress. Although the body can reduce the harmful effects of ER stress through the unfolded protein response (UPR), after SCI, UPR is out of controllable range of protein folding and is not compensated (Ohri et al., 2011). It will stimulate the apoptosis signaling pathway, thereby exacerbating SCI. After accumulation of unfolded protein, it induces the activation of the PERK signal. It will lead to the upregulation of EIF–2α expression, and eventually activate CHOP and other proteins to cause cell apoptosis. Moreover, chaperone proteins such as GRP78 and the caspase 12/caspase 3 pathway will also be activated to induce cell apoptosis (Lee et al., 2014). Studies have shown that SCI can cause numerous neuron deaths, myelin sheath loss, axon damage, and neural circuit defects (Fancy et al., 2011; Sas et al., 2020). Among them, the loss of neurons is an important factor leading to motor dysfunction. Studies have confirmed that a series of biochemical reactions triggered by ER stress may play an important role in neuronal apoptosis after SCI (Springer et al., 1999). Since neurons are difficult to regenerate, reducing their apoptosis becomes an ideal strategy to reduce neuron loss. Therefore, inhibiting the stress of the ER to conserve neurons from apoptosis may be a sensible treatment for SCI.

Pharmacological intervention to inhibit apoptosis has been considered an important direction to reduce neuronal death (Cheng et al., 2018; Song et al., 2019). As an acute stress response, apoptosis mainly occurs in the acute phase of SCI. Consistently, we found that AAV–5HRE–aFGF–NSCs had effectively inhibited the caspase 12/caspase 3 pathway, EIF2α–CHOP pathway, and GRP78 in 7 days, thereby attenuating the apoptosis after SCI and promoting the recovery of the rat’s motor function. After accumulation of unfolded protein, it induces the stimulation of the PERK signaling pathway. The activation of it will increase the expression of EIF–2α/CHOP, leading to cell apoptosis. In addition, the expression of the caspase 12/caspase 3 pathway and GRP78 and other chaperone proteins will also be upregulated, thereby inducing apoptosis. Our research shows that contrasted with the SCI group, the expression of apoptosis-related proteins (such as CHOP, GRP78, and caspase 3) in the NSC-only treatment group was downregulated, which may be owed to the fact that NSCs secrete a small amount of aFGF when stimulated. It has a protective effect on nerve cells, but its expression is far less than the concentration to achieve the best effect, so it needs to be supplemented by exogenous aFGF. Compared with the simple NSCs treatment group, the expression of apoptosis-related proteins in the combined treatment group of aFGF and NSCs is lower, which indicates that the supplementation of exogenous aFGF has a better regulatory effect on ER stress-induced apoptosis.

We used the Basso–Beattie–Bresnahan (BBB) scale and inclined plate test to detect the recovery of the rat’s motor function. In addition, we tested the expression levels of NeuN and apoptosis-related proteins (such as GRP78, caspase 12, and CHOP) induced by ER stress. Data showed that AAV–5HRE–aFGF–NSCs can repair SCI by lessening the ER stress-induced apoptosis.

In short, our research shows that the combined action of aFGF and NSCs protects neurons and promotes the restoration of motor function in SCI rats. This is achieved by reducing apoptosis induced by ER stress.

## Data Availability Statement

The original contributions presented in the study are included in the article/supplementary material, further inquiries can be directed to the corresponding author/s.

## Ethics Statement

The animal study was reviewed and approved by Animal Care and Use Committee of Wenzhou Medical College (wydw2014-0074).

## Author Contributions

YY, YZ, MC, and YT coordinated and carried out most of the experiments and data analysis, and participated in drafting the manuscript. MC, ZH, WY, and QW provided technical assistance. JY and ZX carried out assistance on data analysis and manuscript preparation. SZ and ZW supervised the project and experimental designs and data analysis. SZ, ZW, and XW supervised the project and revised the manuscript. All authors approved the final manuscript.

## Conflict of Interest

The authors declare that the research was conducted in the absence of any commercial or financial relationships that could be construed as a potential conflict of interest.
