# Corrigendum: Hypoxia response element-directed expression of aFGF in neural stem cells promotes the recovery of spinal cord injury and attenuates SCI-induced apoptosis

**DOI:** 10.3389/fcell.2022.1011414

**Published:** 2022-09-13

**Authors:** Yibo Ying, Yifan Zhang, Yurong Tu, Min Chen, Zhiyang Huang, Weiyang Ying, Qiuji Wu, Jiahui Ye, Ziyue Xiang, Xiangyang Wang, Zhouguang Wang, Sipin Zhu

**Affiliations:** ^1^ Department of Orthopaedics, The Second Affiliated Hospital and Yuying Children’s Hospital of Wenzhou Medical University, Wenzhou, China; ^2^ Molecular Pharmacology Research Center, School of Pharmaceutical Science, Wenzhou Medical University, Wenzhou, China; ^3^ Department of Pain Medicine, The Second Affiliated Hospital and Yuying Children’s Hospital of Wenzhou Medical University, Wenzhou, China

**Keywords:** spinal cord injury, acidic fibroblast growth factor, adeno-associated virus, neural stem cell, endoplasmic reticulum stress, apoptosis

In the published article, there was an error in Figure 5 as published. There were similar blocks of EIF-2α and CHOP staining in the AAV-5HRE-NSCs group in Figure 5. The corrected Figure 5 and its caption appear below.

**FIGURE 5:**
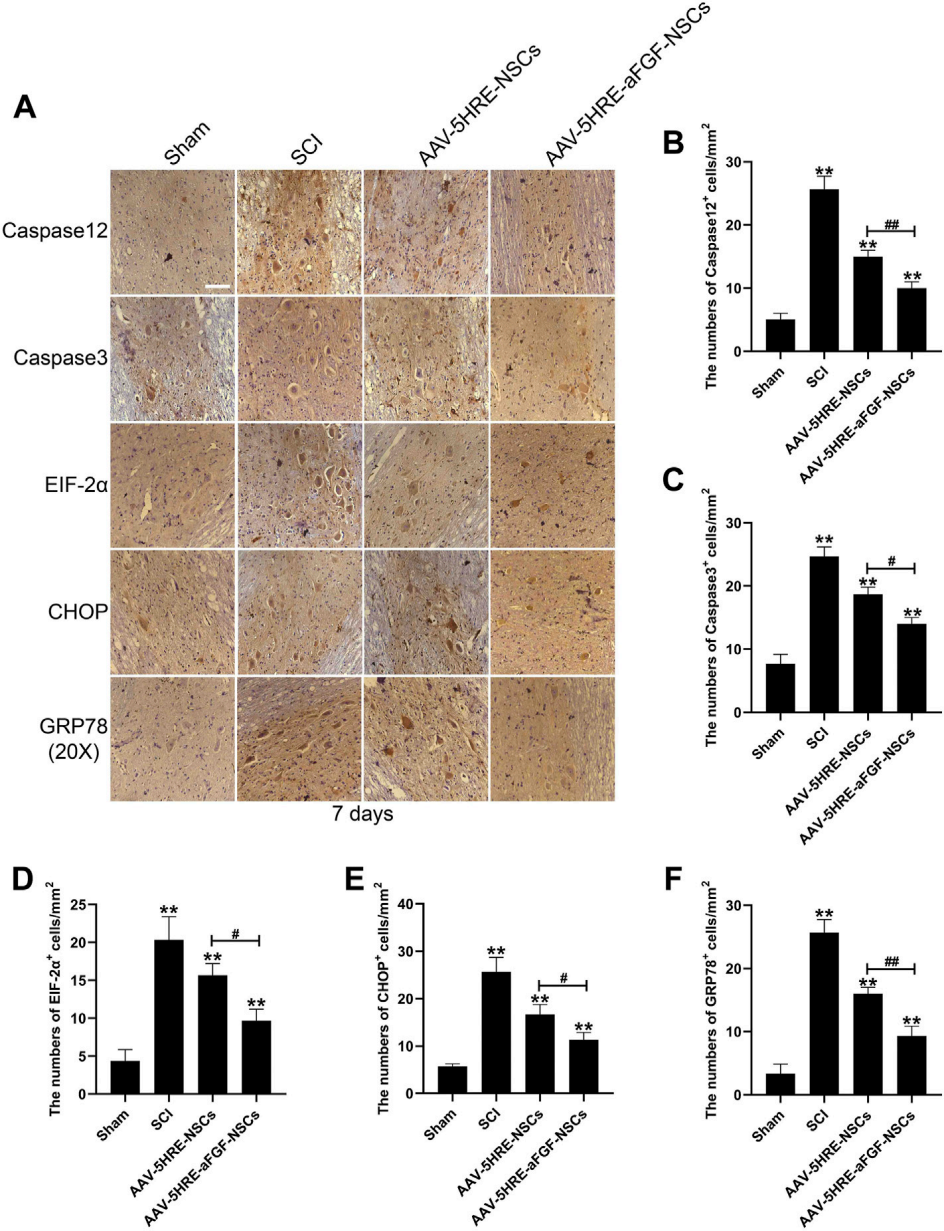
The regulation signals are significant for the neuroprotective effect of AAV–5HRE–aFGF–NSCs. **(A)** Immunohistochemistry for caspase 12, caspase 3, EIF–2a–CHOP, and GRP78 in the Sham, SCI, AAV–5HRE–NSCs, and AAV–5HRE–aFGF–NSCs group. Magnification: 20×; Scale: 100 μm. **(B–F)** Analysis of immunohistochemistry positive cells. **p < 0.01. ^#^ *p* < 0.05. ^##^ *p* < 0.01. Data are represented as mean ± SD (*n* = 6).

The authors apologize for this error and state that this does not change the scientific conclusions of the article in any way. The original article has been updated.

